# In-Hospital Mortality and Associated Factors among Colorectal Cancer Patients in Germany

**DOI:** 10.3390/cancers16061219

**Published:** 2024-03-20

**Authors:** Karel Kostev, Sarah Krieg, Andreas Krieg, Tom Luedde, Sven H. Loosen, Christoph Roderburg

**Affiliations:** 1 Epidemiology, IQVIA, 60549 Frankfurt, Germany; 2 University Clinic, Philipps-University, 35043 Marburg, Germany; 3 Department of Inclusive Medicine, University Hospital Ostwestfalen-Lippe, Bielefeld University, 33617 Bielefeld, Germany; 4 Department of General and Visceral Surgery, Thoracic Surgery and Proctology, University Hospital Herford, Medical Campus OWL, Ruhr University Bochum, 32049 Herford, Germany; 5 Department of Gastroenterology, Hepatology and Infectious Diseases, University Hospital Düsseldorf, Medical Faculty, Heinrich Heine University Düsseldorf, Moorenstrasse 5, 40225 Düsseldorf, Germany

**Keywords:** colorectal cancer, mortality, hospital, database, epidemiology

## Abstract

**Simple Summary:**

With its significant incidence and prevalence, colorectal cancer (CRC) has emerged as a major contributor to both morbidity and mortality. Several studies outside of Germany have investigated the in-hospital mortality of colorectal cancer patients. The present multicenter cross-sectional study includes 4146 hospitalized patients with a main diagnosis of colorectal cancers from 14 hospitals across Germany. The in-hospital mortality rate was 8.7%. Along with higher age, high patient clinical complexity, the presence of distant metastases, renal failure, peritonitis, acute posthemorrhagic anemia, and respiratory failure were associated with an increased risk of mortality. Our findings underscore the critical role of renal failure, peritonitis, acute posthemorrhagic anemia, and respiratory failure in influencing the mortality outcomes of colorectal cancer patients during hospitalization. The awareness and management of these risk factors may guide clinicians in formulating targeted interventions to improve patient outcomes and enhance the quality of care for individuals with colorectal cancer.

**Abstract:**

Background: In the present study, we used the data from 14 hospitals to systematically evaluate the in-hospital mortality of patients with colorectal cancer as well as its influencing factors in Germany. Methods: This multicenter cross-sectional study included hospitalized patients with a main diagnosis of colorectal cancers in the period between January 2019 and July 2023. The outcome of the study was the prevalence of in-hospital mortality. To access the associations between demographic and clinical variables and in-hospital mortality, univariable and multivariable logistic regression analyses were conducted. Results: A total of 4146 colorectal cancer patients (mean age: 70.9 years; 45.3% female) were included. The in-hospital mortality rate was 8.7%. In a multivariable regression, seven variables were significantly associated with an increased in-hospital mortality, including ages of 71–80 years (OR: 2.08; 95% CI: 1.01–4.29), an age group >80 years (OR: 2.44; 95% CI: 1.18–5.05) as compared to an age group ≤ 50 years, patient clinical-complexity level (PCCL) 3 (OR: 3.01 95% CI: 1.81–4.99) and PCCL 4 (OR: 3.76; 95% CI: 2.22–6.38) as compared to PCCL 0, the presence of distant metastases (OR: 4.95; 95% CI: 3.79–6.48), renal failure (OR: 2.38; 95% CI: 1.80–3.14), peritonitis (OR: 1.87; 95% CI: 1.23–2.85), acute posthemorrhagic anemia (OR: 1.55; 95% CI: 1.11–2.15), and respiratory failure (OR: 3.28; 95% CI: 2.44–4.41). Conclusions: Our findings underscore the critical role of renal failure, peritonitis, acute posthemorrhagic anemia, and respiratory failure in influencing the mortality outcomes of colorectal cancer patients during hospitalization. The awareness and management of these risk factors may guide clinicians in formulating targeted interventions to improve patient outcomes and enhance the quality of care for individuals with colorectal cancer.

## 1. Introduction

With its significant incidence and prevalence, colorectal cancer (CRC) has emerged as a major contributor to both morbidity and mortality [1,2]. In Germany, 62,230 new cases and 25,972 deaths were reported in 2018 [1]. The 5-year relative survival rate for colorectal cancer varies from approximately 90% in stage I to 12% in stage IV [3]. A proportion of patients die in hospital, not least due to the complications of surgical or medical therapy. Complications, such as bleeding or ileus, may already be associated with colorectal cancer at the time of diagnosis. The risk of these clinical issues is notably high in elderly and multimorbid patients, comprising the majority of diagnosed individuals. While the prognosis for colorectal cancer patients has notably improved in recent years due to new targeted systemic therapies and the increased use of highly active multimodal treatment approaches, the outlook for multimorbid patients unable to undergo intensive therapies remains unfavorable [4]. Additionally, these regimens are often associated with a range of side effects, leading to significant morbidity, including oxaliplatin-associated polyneuropathy or chronic gastrointestinal complaints resulting from extensive bowel resections.

Several studies have investigated in-hospital mortality in colorectal cancer patients.

Diers et al. evaluated mortality rates following colorectal cancer resection among 129,196 colorectal cancer resections from 2012 to 2015 in Germany. The authors reported a mortality rate of 5.8% [5]. Grewal et al. analyzed a total of 1,962,705 admissions for colorectal cancer in the USA from 2007 to 2017, showing an average in-hospital mortality of 4.9% [6]. In the study by Osler et al. conducted in Denmark in 2001–2004, 30-day in-hospital mortality after emergency colorectal cancer surgery varied from 3.5% to 44.1% [7]. Burns et al. analyzed national data from 33,489 patients who underwent an emergency colorectal resection between 2000 and 2008 and reported a 30-day in-hospital mortality between 6% to 27%, depending on the hospital [8]. 

Although these and other studies are highly relevant, there are still several questions that need to be addressed. First, the majority of studies were conducted outside of Germany, and their findings may not be applicable to the German situation. Second, most of the studies on in-hospital mortality were conducted many years ago, and the mortality rates they report may not reflect recent changes. Third, most studies did not analyze the impact of chronic comorbidities on patient mortality. Considering these limitations, it is clear that more data are needed on the in-hospital mortality of colorectal cancer patients and its determinants in Germany. Therefore, in this study, we systematically investigated this question using data from 14 hospitals in Germany.

## 2. Materials and Methods

### 2.1. Data Source

This multicenter cross-sectional study was based on data from the PREMAX^®^ database (Company: IQVIA (Berlin, Germany)), which contained the §21 dataset of fourteen hospitals across Germany, including specialized hospitals, primary care hospitals, maximum-care hospitals, standard-care hospitals, and university hospitals. The §21 dataset describes a standardized data format that hospitals submit to the InEK (the institute for the payment system in hospitals) in accordance with the §21 KHEntgG (German Hospital Fees Act). The individual treatment episodes included in the §21 dataset of a case is grouped using special grouper software developed by 3M Health Information Systems and IQVIA. In addition, the export files generated by the software are anonymized (e.g., case, and patient number) before transmission for data-protection reasons.

### 2.2. Study Population 

The study included hospitalized patients with a main diagnosis of colorectal cancer (ICD-10: C18, C20) in the period between January 2019 and July 2023. If patients were hospitalized more than one time in the study period, only the last hospitalization was included in the analysis.

### 2.3. Study Outcome

The outcome of the study was the prevalence of the in-hospital mortality. The dataset contains death as one of the discharge types. The proportion of patients who died was calculated for the total population as well as for five age groups (≤50, 51–60, 61–70, 71–80, >80 years), women, men, patients with colon cancer and rectal cancer, as well as for different patient clinical-complexity levels (PCCL; 0—no complications and comorbidity effects, 1—minor complications and comorbidity effects, 2—moderate complications and comorbidity effects, 3—severe complications and comorbidity effects, 4—catastrophic complications and comorbidity effects). 

PCCL is a special index that is calculated for each treatment episode based on a complex algorithm to indicate the effect of complications and comorbidities in a patient [9].

### 2.4. Statistical Analyses 

To access the associations between demographic and clinical variables and in-hospital mortality, univariable and multivariable logistic regression analyses were conducted. The models with mortality (yes, no) as the dependent variable included age groups, sex, cancer type, PCCL, and secondary diagnoses documented in at least 5% of the study population (lymph node metastases (ICD-10: C77), distant metastases (ICD-10: C78, C79), diabetes mellitus (ICD-10: E10–E14), lipid metabolism disorders (ICD-10: E78), hypertension (ICD-10: I10), coronary heart disease (ICD-10: I25), atrial fibrillation and flutter (ICD-10: I48), heart failure (ICD-10: I50), renal failure (ICD-10: N17–N19), thyroid-gland disorders (ICD-10: E00–E07), obesity (ICD-10: E66), paralytic ileus and intestinal obstruction (ICD-10: K56), peritonitis (ICD-10: K65), iron deficiency anemia (ICD-10: D50), acute posthemorrhagic anemia (ICD-10: D62), anemia due to other chronic diseases classified elsewhere (ICD-10: D63), intraoperative and postprocedural complications and disorders of digestive system (ICD-10: K91), and respiratory failure (ICD-10: J96). The results of the logistic regression models were given as the odds ratio (OR) between the analysis and the reference groups (i.e., men versus women, rectal cancer versus colon cancer, etc.). The multivariable model included age, sex, cancer type, all secondary diagnoses, as well as PCCL and was conducted with a stepwise selection to output only variables with significant effects. *p* values < 0.05 were considered statistically significant. All analyses were performed using SAS 9.4 (SAS Institute, Cary, NC, USA).

## 3. Results

### 3.1. Baseline Characteristics 

A total of 4146 colorectal cancer patients were included in the present analysis. In total, 68.6% of admissions were registered as elective care, since 31.4% were registered as emergency care. Of 4146 patients, 64.3% had colon cancer and 35.7% had rectal cancer. The mean age was 70.9 (SD: 12.5) years, and 45.3% of the patients were female. The mean length of a hospital stay was 14.4 (SD: 13.3) days. A total of 31.3% of patients were categorized as PCCL 0 (no complications and comorbidity effects), 13.7% as PCCL 1 (minor complications and comorbidity effects), 12.7% as PCCL 2 (moderate complications and comorbidity effects), 22.4% as PCCL 3 (severe complications and comorbidity effects), and 19.9% as PCCL 4 (catastrophic complications and comorbidity effects). Distant metastases were documented in 25.1% of patients. Hypertension (47.8%), diabetes mellitus (18.0%), and atrial fibrillation/ flutter (14.2%) were the most frequent co-diagnoses (Table 1).

### 3.2. Prevalence of In-Hospital Mortality

**Figure 1:**
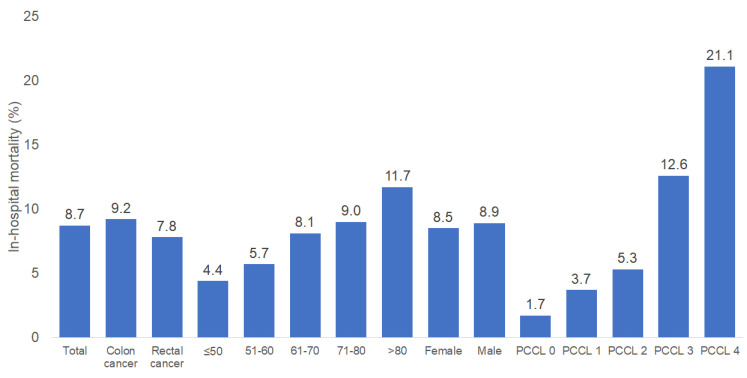
In-hospital-mortality in patients hospitalized for colorectal cancer by cancer type, age, sex, and patient clinical-complexity level. Patient clinical-complexity level: 0—no complications and comorbidity effects, 1—minor complications and comorbidity effects, 2—moderate complications and comorbidity effects, 3—severe complications and comorbidity effects, 4—catastrophic complications and comorbidity effects.

Figure 1 shows the proportion of deceased patients. Of 4146 study patients, 8.7% died. The in-hospital mortality rate was a slightly higher for colon cancer (9.2%) than for rectal cancer (7.8%) and strongly increased from 4.4% in the age group ≤ 50 years to 11.7% in the age group > 80 years. Finally, the in-hospital mortality rate was lowest in patients with PCCL 1 (minor complications and comorbidity effects) (1.7%), and highest in patients with PCCL 4 (catastrophic complications and comorbidity effects) (21.1%).

### 3.3. Factors Associated with In-Hospital Mortality

In the univariable regression model, different variables were associated with an in-hospital mortality, including age groups 71–80, and >80-year-olds versus young patients, PCCL 1,2,3,4 versus PCCL 0, distant metastases, atrial fibrillation and flutter, heart failure, renal failure, paralytic ileus and intestinal obstruction, peritonitis, acute posthemorrhagic anemia, anemia in chronic diseases, and respiratory failure (Table 2). 

However, in a multivariable regression with a stepwise selection, only seven variables were still significantly associated with an increased in-hospital mortality. These included ages 71–80 (OR: 2.08; 95% CI: 1.01–4.29), an age group of >80 (OR: 2.44; 95% CI: 1.18–5.05) compared to an age group of ≤50, PCCL 3 (OR: 3.01 95% CI: 1.81–4.99) and PCCL 4 (OR: 3.76; 95% CI: 2.22–6.38) as compared to PCCL 0, the presence of distant metastases (OR: 4.95; 95% CI: 3.79–6.48), renal failure (OR: 2.38; 95% CI: 1.80–3.14), peritonitis (OR: 1.87; 95% CI: 1.23–2.85), acute posthemorrhagic anemia (OR: 1.55; 95% CI: 1.11–2.15), and respiratory failure (OR: 3.28; 95% CI: 2.44–4.41) (Table 3). 

## 4. Discussion

Here, we present one of the first comprehensive analyses of the clinical presentation, management, and outcome of patients hospitalized for colorectal cancer in Germany. We report an in-hospital mortality rate of 8.7%, which was slightly lower in patients with colon cancer than in those with rectal cancer. Older patients and those with higher a PCCL showed increased in-hospital mortality, which is in line with our findings that comorbidities such as renal or respiratory failure are positively associated with death.

Colorectal cancer, at least in the metastatic stage, is a lethal malignancy. Our cohort consisted of patients hospitalized with a main diagnosis of colorectal cancer. Therefore, we included both patients who were admitted electively for tumor surgery and patients who received elective chemotherapy in the hospital, as well as patients who had to be admitted as an emergency due to the deterioration of their general condition or complications. Nevertheless, the mean age was 70.9 years, and the cohort contained approximately equal proportions of patients in good and poor general health, which certainly reflects the clinical reality in German hospitals very well. Thus, our findings are in line with recent reports, e.g., from the Berlin Cancer Registry or a study reporting nationwide data on in-hospital mortality following colorectal cancer resection in Germany [7,10,11]. 

Unfortunately, our data do not allow us to distinguish between a de novo comorbidity occurring during hospitalization and pre-existing comorbidities. Finally, no data are available on 30- or 90-day mortality rates after hospital discharge, representing an important limitation of the present analysis. Recent data point toward better outcomes for patients treated at high-volume centers, which might be particularly relevant as multimodal therapy strategies, potentially performed with greater experience at high-volume centers, have become increasingly important in CRC. Moreover, high-volume centers offering a higher number of in-house specialists are likely to be better able to care for older and more complex patients. Following the trend toward treatment in specialized centers, the German Cancer Society (Deutsche Krebsgesellschaft) validates cancer treatment centers and requires colorectal cancer centers to meet various criteria, including an annual caseload exceeding 30 patients with colon cancer. Today, approximately 300 centers are certified, collectively conducting about 50% of all colon and rectal cancer resections in Germany [11]. The documented overall 30-day mortality rate after colorectal surgery in certified centers has been reported to be approximately 2.5%. It is important to note that a direct comparison with our present study is not feasible due to the inclusion of nonsurgical and emergency cases in our analysis. 

We show that different comorbidities are associated with in-hospital mortality. Along with older age [12,13,14,15], higher clinical-complexity level, and distant metastases, renal failure, respiratory failure, acute posthemorrhagic anemia, and peritonitis were associated with an increased in-hospital mortality. Importantly, these diagnoses or complications were significantly associated with death after adjustment for clinical-complexity levels.

Respiratory failure is a critical factor contributing to the increased mortality risk in cancer patients. When cancer affects directly or indirectly the respiratory system, it can compromise lung function and lead to respiratory failure, often requiring mechanical ventilation. Cancer-related changes in the lung tissue, such as tumors or inflammation, can hinder the normal respiratory process. Additionally, cancer treatments like chemotherapy and radiation therapy may cause damage to lung tissue, exacerbating respiratory distress. The presence of respiratory failure not only compromises tissue oxygenation but also triggers a cascade of physiological responses that can affect other vital organs. Hypoxia, what is characterized by inadequate oxygen delivery, can lead to organ dysfunction and failure. This multi-organ involvement significantly contributes to the overall mortality risk in cancer patients experiencing respiratory failure. Moreover, the immune system of cancer patients is often compromised and makes them more susceptible to respiratory infections like pneumonia, which can further exacerbate respiratory failure and increase the risk of death. The combination of the underlying cancer, treatment-related complications, and secondary respiratory infections creates a challenging scenario for healthcare providers in hospitals [16,17,18,19].

The occurrence of acute renal failure among cancer patients represents a severe complication associated with significant morbidity and mortality. The pathways leading to acute renal failure in cancer patients share similarities with those observed in other conditions. Nevertheless, acute renal failure can also arise from factors related to cancer treatment, including the use of nephrotoxic chemotherapy agents or the impact of the disease itself. These may involve post-renal obstruction, compression, or infiltration, as well as metabolic or immunological mechanisms [20,21,22].

The observed positive association between acute posthemorrhagic anemia and mortality aligns with the existing literature that suggests that anemia, particularly acute posthemorrhagic anemia, is a critical factor influencing outcomes in cancer patients [23]. Reduced oxygen-carrying capacity, compromised tissue perfusion, and an increased susceptibility to infections and complications may play important roles. Gvirtzman et al. were able to show that anemia may not only be a symptom of highly advanced colorectal cancer, but, the presence of anemia, regardless of its severity, may adversely affect the prognosis during the pre-operative stage [24].

Peritonitis, characterized by the inflammation of the peritoneum, may be a critical complication in colorectal cancer patients, and is associated with a higher mortality risk. The link between peritonitis and increased mortality could be attributed to various factors, such as compromised immune function, delayed diagnosis, or the presence of advanced cancer stages [25].

In addition to the limitations briefly mentioned above, further limitations should be noted. First, both the primary and secondary diagnoses relied exclusively on the ICD-10 classification without accompanying information on symptoms or disease severity. Second, the database used lacked comprehensive details on the chronological order of diagnoses. Third, the lack of medication data precludes an analysis of the potential impact of different drugs on mortality. Fourth, as we analyzed the last hospitalization in patients who were hospitalized more than once in the study period, the morality rate in these patients may be overestimated. Fifth, as the dataset is exclusively derived from hospital records, the outcomes of our study cannot be generalized to the outpatient population. However, the strengths of our study lie in the extensive sample size, the use of data gathered from 14 hospitals across diverse German states, and the comprehensive documentation of comorbidities and mortality information.

## 5. Conclusions

In conclusion, our study of 4146 colorectal cancer patients revealed an overall in-hospital mortality rate of 8.7% with a substantial increase in the mortality rate in higher ages. Our findings underscore the critical role of renal failure, peritonitis, acute posthemorrhagic anemia, and respiratory failure in influencing the in hospital-mortality outcomes of colorectal cancer patients during hospitalization. Recognizing and addressing these risk factors can guide clinicians in formulating targeted interventions to improve patient outcomes and enhance the quality of care for individuals with colorectal cancer.

## Figures and Tables

**Table 1:** Baseline characteristics of the study sample.

Variable	Hospitalized Patients (N = 4146)
Mean age (standard deviation)	70.9 (12.5)
≤50	227 (5.5)
51–60	616 (14.8)
61–70	1004 (24.2)
71–80	1222 (29.5)
>80	1077 (26.0)
Female	1876 (45.3)
Male	2270 (54.7)
Main diagnosis: colon cancer	2666 (64.3)
Main diagnosis: rectal cancer	1480 (35.7)
Mean length of stay in hospital in days (standard deviation)	14.4 (13.3)
Patient clinical-complexity level	
0—No complications and comorbidity effects	1298 (31.3)
1—Minor complications and comorbidity effects	568 (13.7)
2—Moderate complications and comorbidity effects	525 (12.7)
3—Severe complications and comorbidity effects	930 (22.4)
4—Catastrophic complications and comorbidity effects	825 (19.9)
Lymph node metastases	700 (17.1)
Distant metastases	1041 (25.1)
Secondary diagnoses	
Diabetes mellitus	747 (18.0)
Lipid metabolism disorders	555 (13.4)
Hypertension	1983 (47.8)
Coronary heart disease	428 (10.3)
Atrial fibrillation and flutter	589 (14.2)
Heart failure	322 (7.8)
Renal failure	583 (14.1)
Thyroid-gland disorders	522 (12.6)
Obesity	342 (8.3)
Paralytic ileus and intestinal obstruction	510 (12.3)
Peritonitis	220 (5.3)
Iron deficiency anemia	295 (7.1)
Acute posthemorrhagic anemia	397 (9.6)
Anemia in chronic diseases classified elsewhere	495 (11.9)
Intraoperative and postprocedural complications and disorders of the digestive system	408 (9.8)
Respiratory failure, not elsewhere classified	411 (9.9)

Data are absolute numbers (percentages) unless otherwise specified.

**Table 2:** Association of demographic and clinical variables with the in-hospital mortality in patients hospitalized for colorectal cancer (univariable regression model).

Variable	OR (95% CI)	*p* Value
Age groups		
≤50 years	Reference	
51–60 years	1.31 (0.64–2.69)	0.466
61–70 years	1.90 (0.97–3.73)	0.061
71–80 years	2.15 (1–114.17)	0.024
>80 years	2.88 (1.49–5.57)	0.002
Female	Reference	
Male	1.06 (0.85–1.32)	0.596
Main diagnosis: colon cancer	Reference	
Main diagnosis: rectal cancer	0.84 (0.66–1.05)	0.129
Patient clinical-complexity level		
0—No complications and comorbidity effects	Reference	
1—Minor complications and comorbidity effects	2.23 (1.21–4.08)	0.010
2—Moderate complications and comorbidity effects	3.27 (1.85–5.76)	<0.001
3—Severe complications and comorbidity effects	8.34 (5.25–13.27)	<0.001
4—Catastrophic complications and comorbidity effects	15.50 (9.85–24.39)	<0.001
Lymph node metastases	1.09 (0.83–1.44)	0.541
Distant metastases	5.52 (4.41–6.91)	<0.001
Secondary diagnoses		
Diabetes mellitus	1.23 (0.95–1.61)	0.123
Lipid metabolism disorders	0.94 (0.68–1.29)	0.691
Hypertension	0.81 (0.65–1.01)	0.059
Coronary heart disease	1.26 (0.91–1.75)	0.168
Atrial fibrillation and flutter	1.89 (1.46–2.46)	<0.001
Heart failure	2.42 (1.77–3.30)	<0.001
Renal failure	4.37 (3.45–5.52)	<0.001
Thyroid-gland disorders	1.21 (0.89–1.65)	0.219
Obesity	0.63 (0.40–1.00)	0.051
Paralytic ileus and intestinal obstruction	2.70 (2.08–3.49)	<0.001
Peritonitis	4.04 (2.92–5.59)	<0.001
Intraoperative and postprocedural complications and disorders of the digestive system	1.34 (0.96–1.86)	0.084
Iron deficiency anemia	1.35 (0.92–1.97)	0.122
Acute posthemorrhagic anemia	3.10 (2.36–4.08)	<0.001
Anemia in chronic diseases classified elsewhere	1.60 (1.20–2.14)	0.002
Respiratory failure, not elsewhere classified	6.35 (4.95–8.14)	<0.001

**Table 3:** Association of demographic and clinical variables with the in-hospital mortality in patients hospitalized for colorectal cancer (multivariable regression model).

Variable	OR (95% CI) *	*p* Value
Age groups		
≤50 years	Reference	
71–80 years	2.08 (1.01–4.29)	0.047
>80 years	2.44 (1.18–5.05)	0.016
Patient clinical-complexity level		
0—No complications and comorbidity effects	Reference	
3—Severe complications and comorbidity effects	3.01 (1.81–4.99)	<0.001
4—Catastrophic complications and comorbidity effects	3.76 (2.22–6.38)	<0.001
Distant metastases	4.95 (3.79–6.48)	<0.001
Secondary diagnoses		
Renal failure	2.38 (1.80–3.14)	<0.001
Peritonitis	1.87 (1.23–2.85)	0.004
Acute posthemorrhagic anemia	1.55 (1.11–2.15)	0.009
Respiratory failure, not elsewhere classified	3.28 (2.44–4.41)	<0.001

* Multivariable regression model 2: stepwise selection of variables; adjusted for age, sex, cancer type, all secondary diagnoses, and patient clinical-complexity level.

## Data Availability

The datasets used and analyzed during the current study are available from the corresponding author on reasonable request.
